# Diagnoses of postpartum urinary retention using next-generation non-piezo ultrasound technology: assessing the accuracy and benefits

**DOI:** 10.1038/s41598-024-83160-6

**Published:** 2024-12-30

**Authors:** Ruben Plöger, Charlotte Behning, Adeline Walter, Ulrich Gembruch, Brigitte Strizek, Florian Recker

**Affiliations:** 1 Department of Obstetrics and Prenatal Medicine, University Hospital Bonn, Venusberg Campus 1, 53127 Bonn, Germany; 2 Institute for Medical Biometry, Informatics and Epidemiology, University Hospital Bonn, Venusberg Campus 1, 53127 Bonn, Germany

**Keywords:** Urology, Obstetrics, Personal-device-based-point-of-care-ultrasound, Point-of-care-ultrasound, POCUS, Semiconductors, Piezo-based, Chip-based, Urinary retention, Postpartum, Medical imaging, Outcomes research, Clinical trial design

## Abstract

Postpartum urinary retention has a wide range of publicized incidences, likely caused by frequent misdiagnosis of this puerperal complication. Especially covert postpartum urinary retention has a high number of missed diagnoses due to the lack of symptoms and the time-extensive diagnostics via ultrasound, leading to no treatment and no appropriate follow-up. To simplify the diagnosis and establish a screening tool we analyzed the application of portable handheld-ultrasound devices  (PUD) as used in Point-of-care diagnostics in comparison to established standard ultrasound devices (SUD). This prospective study aimed to evaluate the reliability of non-piezo, chip-based PUD in comparison to the measurement withSUD, containing a piezo transducer, as golden standard for the ultrasound diagnosis of postpartum urinary retention. Randomly, 100 participants between the first and seventh day after delivery in an obstetric ward underwent ultrasound examinations using a EPIQ 5 W (Philips) as SUD and a Butterfly iQ (Butterfly Network) as PUD to compare the accuracy in bladder size after micturition and the estimated post-void residual volume. Intraclass correlation coefficients, Bland-Altman plots, and Pearson correlation coefficients were used for analyzing the reliability and agreement between the measurements of these devices and were calculated for subgroups as body mass index, mode of delivery and timepoint of delivery. The results show a near-perfect agreement (0.994) and correlation (*r* = 0.982) for estimated post-void residual volume and for most measurements between the two types of ultrasound devices. The agreement rate for the diagnosis of covert postpartum urinary retention is 100%. Subgroup analyses lack a significant difference reflected by agreement and correlation rates. These findings affirm the high reliability of PUD for the diagnosis of postpartum urinary retention and supports their integration into daily clinical practice, thereby simplifying regular controls of the bladder by physicians during daily rounds on the ward. This technology may allow a higher diagnosis rate so that patient care can be optimized and the long-term impact on continence and quality of life can be studied and analysed.

## Introduction

Postpartum urinary retention (PUR) is a common complication after childbirth with an incidence varying from 0.18 to 20.6%^1–4^. As common definition PUR is divided into overt and covert retention^5^: Overt PUR is defined as an inability to urinate 6 h after vaginal delivery or after catheter removal following caesarean section. Covert PUR is defined as a post-void residual bladder volume of more than 150 ml in post-partum patients without urinary symptoms. . In several studies^6^, however, overt PUR is analyzed using other definitions such as the inability to undergo spontaneous micturition within 12 h after vaginal delivery^7^ or the requirement of at least one catheterization within the first 24 h postpartum^8,9^. Women requiring intermittent catheterization after PUR have long-term voiding difficulties, as reported by 5% of women within three years after diagnosis^10^. An early diagnosis and timely intervention improves the outcome^1^ so that these patients would benefit from an improved diagnostic tool, especially in the case of covert PUR with its high estimated number of unknown cases (approx. nine times the rate of overt PUR^4^). The measurement of post-void residual volume (PVRV) using a urinary catheter as gold standard^11^ is time- and staff-expensive as well as unpleasant for the patient. A measurement of PVRV via ultrasound is reported as highly reliable in most studies^28,29^ but is - at least - accurate enough for clinical decision marking despite the ongoing discussion regarding the exact formula for the calculation of the PVRV in comparison to the measurement using the catheter^12^. A sonographic monitoring^13^ of all postpartum patients with standard ultrasound devices (SUD) is still time- and staff-expensive so that no screening program for PUR has been established so far.

The next-generation of ultrasound machines consists of transducers developed with non-piezo silicon chip technology and connected to a smart-phone or tablet making them easily transportable. This new application of portable ultrasound devices (PUD) enables physicians to perform bedside examinations without heavy transport of the established SUD^14^. Therefore, the use of PUD may lead to a low threshold for an examination of the bladder, especially during daily rounds. While the applications of the PUD have been reported in several areas of obstetrics and gynecology^15^, such as examinations of early pregnancy, of gynecological pathology^16^, of postpartal complications regarding the uterus^17^ and of fetal biometry^16,18–21^, the application of the PUD for examinations of the bladder and exclusion of a PUR has not been assessed thus far in regards to its reliability and agreement.

The aim of this study is to assess the reliability and agreement between measurements of bladder dimensions and calculated PVRV obtained using both SUD and PUD in postpartum patients. It aims to determine whether PUD can serve as a reliable tool for diagnosing PUR and assessing bladder dimensions in postpartum patients, potentially offering a more accessible and cost-effective alternative to traditional SUD.

## Methods

**Fig. 1:**
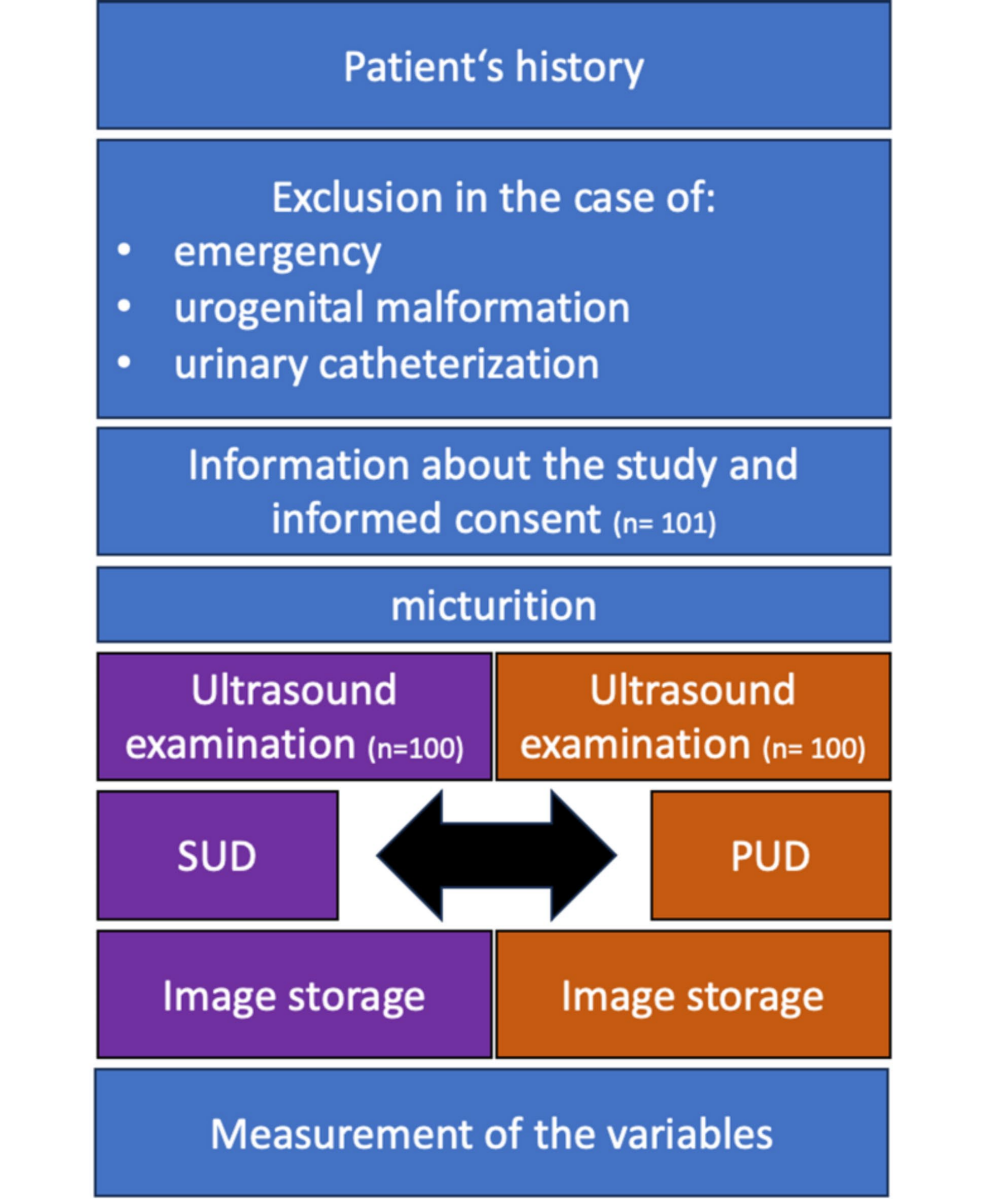
Flow chart of the study design. Number (n). Standard ultrasound device (SUD), Portable ultrasound device (PUD).

**Fig. 2:**
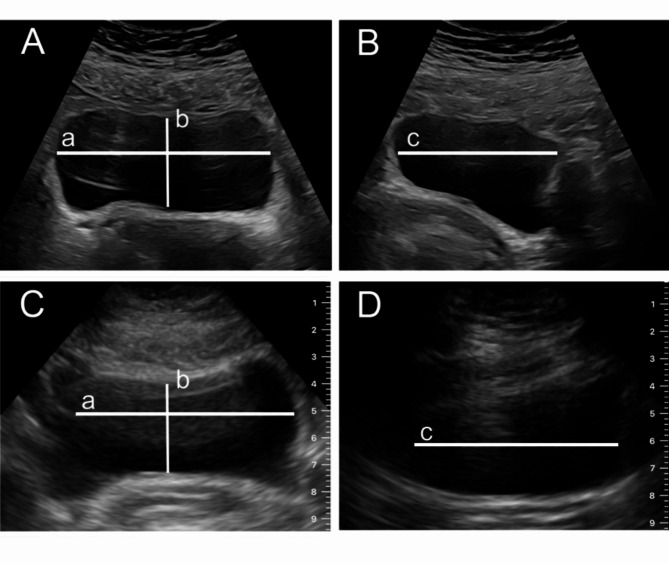
Images of transabdominal ultrasound scans using a standard ultrasound device based on piezo technology (**A**, **B**) and a portable ultrasound device based on silicon chips (**C**, **D**): Demonstration of the transversal scan plan (**A**, **C**) showing the horizontal axis (**a**) and the sagittal axis (**b**) and of a sagittal scan plan (**B**, **D**) showing the longitudinal axis (**c**).

A prospective, observational investigation was carried out over a four-month interval (August to November 2023) within the Department of Obstetrics at a tertiary-level university hospital. The patients were informed about study participation and provided informed consent. The study was approved by the University Hospital Bonn’s Ethics Board (No. 345/21). The study was performed in accordance with the Declaration of Helsinki. Eligibility for participation was the in-patient care on the postnatal ward and, thus, included patients mostly between the first and third day after delivery. The treatment of patients with complications such as neonatal hyperbilirubinaemia or puerperal mastitis at this ward allowed the inclusion of patients with a longer period after delivery, as well. The examination was only performed once on each participant and was realized randomly on one day during their stay. The examinations were always perfromed prior to the participants’ discharge. Emergent patients and those with urogenital malformations were excluded. Patients with temporary urinary catheterization after cesarean section were asked to participate six hours after catheter removal. Additionally, the operational capacity of the Department of Obstetrics to facilitate precise dual-ultrasound device examinations was a prerequisite. Therefore, patients were randomly asked to participate when the operational capacity allowed the examinations. The patients’ medical histories were either acquired during their first presentation in the delivery room prior to delivery or acquired by the ward physicians at the postnatal admission in the case of puerperal complications. During daily rounds the patients were informed about the study’s objectives and methodology. After informed consent the participants were asked about their micturition in the 6 h after delivery to identify overt PUR and then asked to urinate prior to the examination. The investigative protocol entailed the random application of both PUD and SUD for capturing images of predetermined variables prior to measuring in order to mitigate bias (Fig. 1). A final catheterization after the micturition and examination to compare the post-void residual volume (PVRV) with the calculated volume based on the ultrasound diagnostic were not performed to prevent harm such as possible infection or pain for the participants. Every participant underwent a unique examination with a SUD (EPIQ 5 W by Philips, Amsterdam, Netherland) and a PUD (Butterfly iQ by Butterfly Network, Guilford, Connecticut, USA), conducted by the same operator. The SUDs are based on the piezoelectric crystal transducer and the PUD on a silicon chip using the conversion of voltage to resonance. The order of the captured images was standardized during the use of each ultrasound machine, thereby maximizing the period between each image of the same variable and reducing memory bias (Fig. 1). These assessments were carried out either by an assistant physician, trained in obstetrical scanning for two years, or consultants, trained in obstetrical scanning for more than two and less than ten years. Four examiners were part of this study (RP, AW, BS, FR). The collected data including the demographic information of patients’ histories wasrecorded and stored on the university hospital’s server using Excel software (Microsoft Corp., Redmond, WA, USA). Biometric measurements of the bladder were determined (Fig. 2), and the PVRV was calculated using the following formula:

PVRV (ml) = horizontal axis of the bladder (HA) (cm) X longitudinal axis of the bladder (LA) (cm) X sagittal axis of the bladder (SA) X 0.7 (cm)^22^.

A calculated post-void residual bladder volume of more than 150 ml defined covert PUR in this study^5^.

The study’s primary endpoint was the concordance of PVRV estimation, with secondary endpoints including HA, LA and SA of the bladder and the agreement rate for overt and covert PUR.

To avoid confirmation bias, measurements were performed post-image acquisition with each device, with a standardized sequence for PUD and SUD assessments ensuring a consistent interval between measurements of the same variable. Cases with incomplete data were excluded (*n* = 1), and the study concluded upon recruiting 101 patients, so that ultimately 100 patients were included (Fig. 1).

### Statistical methods

The sample sizes of 100 participants was determined based on clinical feasibility and comparable studies^19^ so that the results of this pilot study are descriptive^23^. Statistical analyses and graphical representations were conducted using the Statistical Package for the Social Sciences (SPSS) software, version 27 (IBM Corp., Armonk, NY, USA). To evaluate the consistency and reliability of measurements derived via SUD and PUD, several statistical metrics were employed: Pearson correlation coefficient (PCC) with a 95% confidence interval using Wald methods, intraclass correlation coefficient (ICC) employing a two-way random-effect, agreement model with a 95% confidence interval, and Bland-Altman plots ^24^. An ICC and a PCC approaching the value of 1.0 indicate a high degree of agreement or correlation, as supported by the literature^25–27^. Bland-Altman plots were specifically utilized to qualitatively assess the agreement between measurement methodologies. The mean relative difference (MRD) was calculated by taking the absolute difference between individual measurements and the aggregate mean of these measurements, summing these differences for all subjects, and then dividing by the total number of cases. Subgroup categorization was based on mode of delivery, body mass index and day after delivery. The examiners were asked for feedback by completing a questionnaire.

## Results

A total of 100 paired postpartum ultrasound scans were performed from August to November 2023 (Fig. 2). The demographic characteristics of the study participants are outlined in Table 1. Age of the participants was between 18 and 45. 56% of participants delivered their first child, 32% delivered their second child, 10% delivered their third child and 2% delivered their fourth child. The child was delivered by spontaneous vaginal birth in 57%, by vacuum extraction in 15% and by caesarean section in 28% of the cases. The cause of caesarean section was elective in 39% and emergent in 61%. Preconceptional body mass index (BMI) varied from 16.9 to 37.9 kg/m^2^. Analysis of BMI distribution revealed that a majority, 62%, had a BMI within the normal range (18.5 to < 25 kg/m^2^), whereas 4% were categorized as underweight (BMI < 18.5 kg/m^2^). Notably, 34% of the cohort was identified as overweight (BMI ≥ 25 kg/m^2^); within this group, 53% had a BMI between 25 to < 30 kg/m^2^, 38% fell into obesity class 1 (BMI 30 to < 35 kg/m^2^) and 8% into obesity class 2 (BMI 35 to < 40 kg/m^2^). Of the scans, 43%, 33%, 12%, 3%, 4%, 1% and 2% were performed on day 1, 2, 3, 4, 5, 6 and 7 postpartum, respectively.

**Table 1:** Patients characteristics. number (n)

Patients’ characteristics	*n* = 100
Maternal age (year)^a^	32.8 (5.02)
Gravidity^b^	2 (1–12)
Parity^b^	1 (1–4)
Primiparous^c^	56
Multiparous^c^	44
Gestational age at delivery (week + day)^b^	39 + 3 (30 + 3 – 41 + 5)
Spontaneous vaginal delivery^c^	57
Vacuum-assisted vaginal delivery^c^	15
Caesarean delivery^c^	28
Elective Caesarean delivery^c^	11
Emergency Caesarean delivery^c^	17
Body mass index (kg/m^2^)^a^	24.7 (4.41)
Underweight^c^	4
Normal weight^c^	62
Overweight and Obesity^c^	34
Overweight^c^	18
Obesity class 1^c^	13
Obesity class 2^c^	3
Obesity class 3^c^	0

^a^mean (± standard deviation).

^b^median (range).

^c^number.

In this study no overt PUR was reported because all participants were able to micturate. Five cases of covert PUR were diagnosed using PUD and SUD and confirmed by measurement of the PVRV. Thus, the two methods had an agreement rate of 100% for the diagnoses (Table 2). The mean and standard deviation of the examinations with the established standardized ultrasound device are reported dependent on mode of delivery, on the body mass index and the day of scan, and show a slight difference (s. Table 2).

**Table 2:** Results of the scan dependent on mode of delivery, body mass index, day of the scan using standard ultrasound device (SUD (straight)) using piezo technique and portable ultrasound device (PUD (*italic*)) using silicon chip technique: covert postpartum urinary retention (cPUR), Caesarean delivery (CD), horizontal axis of the bladder (HA), longitudinal axis of the bladder (HA), number (n), normal delivery (ND), normal weight (NW), overt postpartum urinary retention (oPUR), overweight (OW, including obesity), portable ultrasound device (PUD), postpartum urinary retention (PUR), estimated post-void residual volume (PVRV), sagittal axis of the bladder (SA), scan at the first day postpartum (S1PP), scan at the second day postpartum (S2PP), standard ultrasound device (SUD), spontaneous vaginal delivery (SVD), underweight (UW, ), vacuum-assisted vaginal delivery (VAVD).

	SVD, *n* = 57	VAVD, *n* = 15	CD, *n* = 28	UW, *n* = 4	NW, *n* = 62	OW, *n* = 34	S1PP, *n* = 43	S2PP, *n* = 33	Total, *n* = 100
HA (cm)^a^	3.45 (2.66)	0.88 (2.59)	1.23 (2.85)	0.26 (3.76)	3.66 (2.79)	1.70 (2.53)	6.05 (2.47)	5.29 (13.22)	5.62 (2.75)
*HA (cm)* ^*a*^	*3.57 (2.65)*	*0.90 (2.78)*	*1.32 (2.87)*	*0.27 (3.98)*	*3.76 (2.80)*	*1.77 (2.60)*	*6.16 (2.47)*	*5.52 (3.37)*	5.80 (2.79)
SA (cm)^a^	1.58 (1.68)	0.34 (1.49)	0.62 (1.79)	0.14 (2.27)	1.62 (1.68)	0.78 (1.64)	2.53 (1.60)	2.53 (1.94)	2.54 (1.69)
*SA (cm)* ^*a*^	*1.58 (1.70)*	*0.33 (1.37)*	*0.60 (1.74)*	*0.13 (2.01)*	*1.64 (1.66)*	*0.74 (1.65)*	*2.55 (1.57)*	*2.58 (1.94)*	2.52 (1.68)
LA (cm)^a^	1.24 (1.35)	0.34 (1.86)	0.57 (1.77)	0.10 (1.63)	1.45 (1.75)	0.59 (0.99)	2.13 (1.49)	2.08 (1.63)	2.15 (1.54)
*LA (cm)* ^*a*^	*1.28 (1.37)*	*0.32 (1.86)*	*0.57 (1.74)*	*0.10 (1.60)*	*1.48 (1.73)*	*0.59 (1.03)*	*2.08 (1.37)*	*2.17 (1.67)*	2.17 (1.54)
PVRV (ml)^a^	24.67 (57.69)	5.31 (57.01)	8.84 (40.88)	2.84 (89.54)	27.37 (56.67)	8.61 (38.05)	37.83 (50.94)	43.23 (64.78)	38.82 (53.10)
*PVRV (ml)* ^*a*^	*26.23 (58.70)*	*5.23 (52.73)*	*9.00 (41.81)*	*2.88 (83.91)*	*28.84 (57.43)*	*8.73 (38.40)*	*37.40 (49.51)*	*47.29 (65.69)*	40.45 (53.62)
oPUR^b^ (n)	0	0	0	0	0	0	0	0	0
*oPUR* ^b^ *(n)*	*0*	*0*	*0*	*0*	*0*	*0*	*0*	*0*	*0*
cPUR^b^ (n)	3	1	1	1	3	1	3	2	5
*cPUR* ^b^ *(n)*	*3*	*1*	*1*	*1*	*3*	*1*	*3*	*2*	*5*

^a^mean (± SD).

^b^number.

**Fig. 3:**
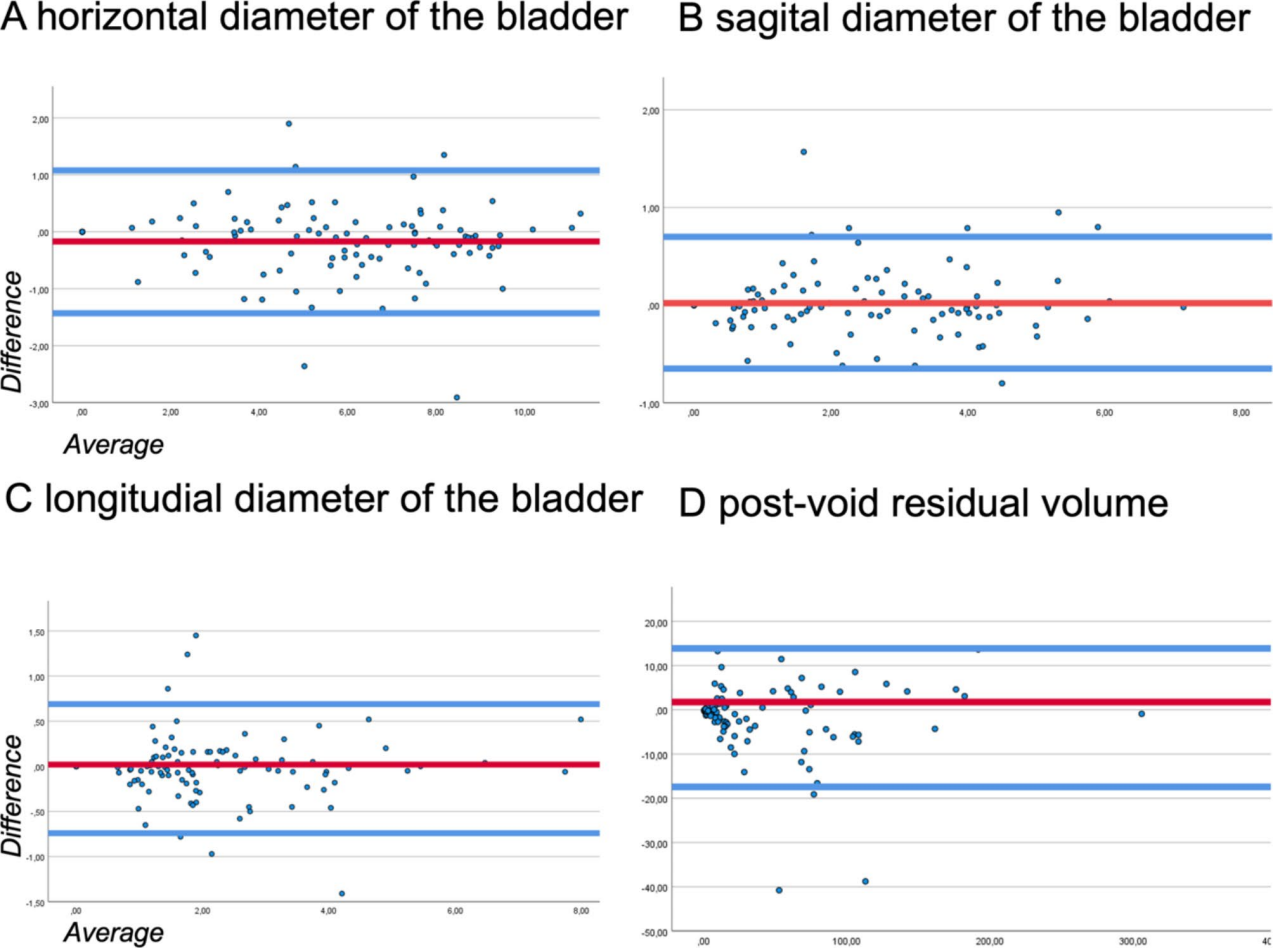
Bland-Altman analysis of the horizontal axis of the bladder (HA), the sagittal axis of the bladder (SA), the longitudinal axis of the bladder (LA) and the post-void residual volume (PVRV) between the devices: standard ultrasound device with piezo technology and portable ultrasound device with silicon chip technology.

The Bland-Altman analysis (Fig. 3; Table 3) for HA, SA, LA and PRV showed a similar performance between SUD and PUD with an average difference of -0.177, 0.021, -0.023 and − 1.751, respectively (s. Table 3). The 95% limits of agreement were between − 0.647 cm and 0.690 cm in the case of the HA’s measurement, -0.647 cm to 0.689 cm in the case of the SA’s measurement, -0.734 cm to 0.687 cm in the case of the LA’s measurement and − 17.404 to 13.899 in the case of the calculated PRV.

The ICC (Table 3) values showed an excellent agreement for the measurements of the HA, SA and LA resulting in an excellent agreement for the PRV^27^. The PCC indicated near-perfect correlation for the measurements of HA, SA and LA as well as for the calculated PRV. The mean relative difference of the HA, SA and LA and PRV was 9%, 12%, 12% and 20%, respectively.

**Table 3:** Average difference, Bland-Altman 95%-limits of agreement, intraclass correlation (ICC, 95% confidence interval) and Pearson correlation coefficient (PCC, 95% confidence interval using Wald methods) and the mean relative difference (MRD) of horizontal axis of the bladder (HA), sagittal axis of the bladder (SA), longitudinal axis of the bladder (LA) and post-void residual volume (PVRV) between the devices: standard ultrasound device using piezo technic and portable ultrasound device using silicon chip technic.

	Average difference	95%-Limits of agreement in cm	ICC (95%)	PCC (95%)	MRD
HA (cm)	-0,177	-0.647 to 0.690	0.986 (0.978–0.991)	0.973 (0.927–1.019)	9%
SA (cm)	0.021	-0.647 to 0.689	0.990 (0.985–0.993	0.980 (0.939–1.020)	12%
LA (cm)	-0.023	-0.734 to 0.687	0.986 (0.979–0.991)	0.973 (0.926–1.020)	12%
PVRV (ml)	-1.751	-17.404 to 13.899	0.994 (0.991–0.996)	0.982 (0.952–1.012)	20%

Upon examining the influence of mode of delivery, the ICC (Table 4) and the PCC (Table 5) showed a high agreement and correlation for all modes of delivery. Regarding the influence of the body mass index (BMI) the subgroup normal weight range (18.5 to < 25 kg/m^2^) with 62 participants and the subgroup overweight (≥ 25 kg/m^2^) with 34 participants were analyzed, whereas the subgroup underweight (< 18.5 kg/m^2^) included only 4 participants and thus was too small for an analysis. The subgroups normal weight and overweight showed similar ICC except for the measurement of longitudinal axis where the confident intervals of the ICC lacked an overlap. However, both ICCs still indicate an excellent reliability^27^. The PCC indicate a high reliability and – as the ICC – showed no great differences between the measurements. The final subgroup analyses concerned the timepoint of examination: ICC and PCC proved an excellent agreement and reliability for examination at the first and second day after delivery (Tables 4 and 5).

**Table 4:** Subgroup analysis of the intraclass correlation coefficients (ICC) with 95%-confidence interval for agreement between measurements from standard ultrasound device and portable ultrasound device for the horizontal axis of the bladder (HA), longitudinal axis of the bladder (LA), sagittal axis of the bladder (SA) and post-void residual volume (PVRV). Caesarean delivery (CS), number (n), normal weight (NW), overweight (OW, including obesity), scan at the first day postpartum (S1PP), scan at the second day postpartum (S2PP), spontaneous vaginal delivery (SVD), vacuum-assisted vaginal delivery (VAVD).

	SVD, *n* = 57	VAVD, *n* = 15	CD, *n* = 28	NW, *n* = 62	OW, *n* = 34	S1PP, *n* = 43	S2PP, *n* = 33	Total, *n* = 100
HA (cm)	0.982 (0.967–0.989)	0.980 (0.941–0.993)	0.994 (0.987–0.997)	0.982 (0.970–0.989)	0.990 (0.979–0.995)	0.981 (0.965–0.990)	0.990 (0.979–0.995)	0.986 (0.978–0.991)
SA (cm)	0.993 (0.988–0.996)	0.991 (0.975–0.997)	0.983 (0.965–0.992)	0.991 (0.984–0.994)	0.988 (0.975–0.994)	0.989 (0.980–0.994)	0.996 (0.992–0.998)	0.990 (0.985–0.993
LA (cm)	0.981 (0.968–0.989)	0.990 (0.972–0.997)	0.991 (0.981–0.996)	0.990 (0.984–0.994)	0.961 (0.921–0.980)	0.986 (0.975–0.993)	0.994 (0.988–0.997)	0.986 (0.979–0.991)
PRV (ml)	0.994 (0.989–0.996)	0.998 (0.994–0.999)	0.994 (0.987–0.998)	0.994 (0.990–0.997)	0.994 (0.988–0.997)	0.998 (0.995–0.999)	0.995 (0.985–0.998)	0.994 (0.991–0.996)

**Table 5:** Subgroup analysis of the Pearson correlation coefficient (PCC) with a 95% confidence interval using Wald methods between measurements from standard ultrasound device and portable ultrasound device for the horizontal axis of the bladder (HA), longitudinal axis of the bladder (LA), sagittal axis of the bladder (SA) and post-void residual volume (PRV). Caesarean delivery (CD), number (n), normal weight (NW), overweight (OW, including obesity), scan at the first day postpartum (S1PP), scan at the second day postpartum (S2PP), spontaneous vaginal delivery (SVD), vacuum-assisted vaginal delivery (VAVD).

	SVD, *n* = 57	VAVD, *n* = 15	CD, *n* = 28	NW, *n* = 62	OW, *n* = 34	S1PP, *n* = 43	S2PP, *n* = 33	Total, *n* = 100
HA (cm)	0.967 (0.897–1.036)	0.962 (0.797–1.126)	0.988 (0.927–1.048)	0.966 (0.900-1.033)	0.983 (0.918–1.049)	0.963 (0.877–1.049)	0.984 (0.919–1.049)	0.973 (0.927–1.019)
SA (cm)	0.986 (0.940–1.031)	0.986 (0.885–1.086)	0.967 (0.869–1.066)	0.981 (0.932–1.031)	0.977 (0.900-1.054)	0.978 (0.913–1.044)	0.993 (0.948–1.037)	0.980 (0.939–1.020)
LA (cm)	0.964 (0.891–1.036)	0.980 (0.862–1.099)	0.981 (0.907–1.056)	0.981 (0.930–1.031)	0.923 (0.785–1.062)	0.977 (0.908–1.045)	0.990 (0.939–1.042)	0.973 (0.926–1.020)
PRV (ml)	0.988 (0.947–1.030)	0.998 (0.955–1.040)	0.989 (0.925–1.053)	0.989 (0.949–1.029)	0.988 (0.931–1.045)	0.995 (0.964–1.026)	0.992 (0.941–1.043)	0.982 (0.952–1.012)

The evaluation of the examiners was mostly positive, as describedd in Table 6.

**Table 6:** Feedback of the examiners regarding the new ultrasound device.

Positive feedback	Negative feedback
Simple handling	Little screens depending on device (tablet versus smart phone)
Time saving	Empty battery
Low threshold for indication of examination	
Adequate solution	
Examination close to the patient	

## Discussion

This prospective investigation for the measurement of the bladder examined the compatibility and reliability of SUD and a PUD. Employing statistical analysis tools such as the PCC, ICC and Bland-Altman plot, this study demonstrated a high correlation and agreement of the measurements with both ultrasound devices.

Concerning the diagnosis of covert postpartum urinary retention on basis of the examinations with both devices, the similar diagnostic results indicate the applicability of the new ultrasound device. The use of PUD would allow a rapid identification of critical patients, and the suspected diagnosis could be verified by measuring the PVRV by catheterization as gold standard without the further use of a PUD. The accuracy of PVRV using transabdominal ultrasound is reported as high in most studies^28,29^ but depends on the used formula^22^. The validation of the PUD’s PVRV estimation via urinary catheterization could be tested in a high-powered study, in which the diagnosis of more than the reported five cases of covert PUR is likely. A comparison between the the calculated PVRV and the measured PVRV via urinary catheterization for these five cases would lack a statistical relevance. Thus, a high-powered study could add a new perspective of the ongoing discussion of the formula and of the incidence of PUR. Therefore, this study focused on the validation of the new ultrasound device in comparison to the established diagnostic methods.

The ICC shows an excellent agreement with 0.986 for HA, 0.99 for SA and 0.986 for LA and thus reveal better results than the known inter-rater ICC determined in urological ultrasound and are comparable with the intra-rater ICC, despite different ultrasound devices^30^. The result of the ICC for the estimated PVRV indicates an excellent agreement and is in harmony with the results of the ICC in other obstetric ultrasound examinations^16–21^. Other markers for analyzing the agreement between the two different assays, such as the PCC and the Bland-Altman plot, also show an excellent agreement and support the results of the ICC. The analysis of possible confounding factors such as obesity, mode of delivery and the timepoint of examination reveals that the new generation of ultrasound devices still shows a high agreement and reliability with the SUD, independent of these factors. In other obstetric ultrasound examinations the BMI influences the ICC due to the varying distance between the PUD transducers, with their smaller acoustic windows than the SUD transducers, and the object of interest^17^.

The feedback of the examiners highlights the simple handling and the time efficiency of the PUD in comparison to the SUD. This resulted in a low threshold to indicate an examination and thus may allow a better care of the patient. The resolution (Fig. 2) was reported to be sufficient for the examination despite the higher resolution of the SUD, suiting the high agreement and reliability of the measurements. The examiners further mentioned the patient benefits from a bedside examination. Negative feedback reflected the small screen, in the case of using a cellphone instead of a tablet, although no measurable effect on the reliability was determined. The problem of empty batteries could be prevented by appropriate charging.

This reliability study opens doors for new diagnostic opportunities, including the comprehensive diagnostic confirmation of in-patient postpartum urinary retention, or as an easy diagnostic tool for out-patient cases or for patients in underdeveloped regions without access to SUD. The first data regarding the feasibility and reliability of self-measurement of PVRV are promising^31^ and may enable the establishment of self-measurement for follow-ups, simplifying the investigation of long-term symptoms^32^. The use of new generation handheld ultrasound devices further enables a diagnostic tool to ascertain the incidence, to understand the development and the impact of PUR.

## Limitations

Several limitations are inherent in this study design and scope that merit consideration: Primarily, the investigation’s single-center approach, conducted exclusively at a tertiary-level university hospital, potentially narrows the applicability of its findings. The unique environment and patient demographic of a single institution may not reflect the diverse settings and populations encountered in various healthcare systems, thus limiting the generalization of the results to broader clinical practices.

Furthermore, the study’s participant selection criteria led to unbalanced subgroups, notably in terms of body mass index, mode of delivery, and investigation period among the participants. This imbalance affects the ability to accurately assess the impact of these factors on the performance and reliability of PUDs compared to SUDs and thus could skew the analysis. The study was performed with one portable ultrasound system with a non-piezo, chip-based technology. Almost every ultrasound manufacturer has developed their own portable, handheld ultrasound device by now. Each device has its own technical specifications and works on different platforms and apps, so that results from one device cannot be generalized for all other PUDs. Aspects such as patient satisfaction, cost-effectiveness, and the potential for enhancing access to prenatal monitoring in underserved communities were not explored in our study. Such considerations are crucial for assessing the holistic value of integrating PUDs into general obstetric care.

Lastly, the study does not include any longitudinal follow-up to evaluate the long-term impacts of using PUDs on clinical outcomes or decision-making in patient management. Incorporating such follow-up could provide invaluable insights into the efficacy and practical benefits of PUD integration into routine obstetric care practices.

## Conclusion

Postpartum urinary retention is a common complication whose established diagnostic tools unfortunately have relevant limitations: ultrasound examinations with standard devices are time-consuming leading to delayed diagnosis, and urinary catheterization is an invasive procedure with risk of further complications. This study presents a new diagnostic tool with the new generation of handheld ultrasound, allowing a point-of-care examination in a time-efficient manner during daily rounds. The agreement and reliability of the ultrasound examination is high in comparison to the established ultrasound examination, so that the application allows precise diagnostics and may help determine the real incidence and impact of postpartum urinary retention in the patients’ life.

## Data Availability

The datasets used and/or analysed during the current study available from the corresponding author on reasonable request.
